# The Effect of *Lycii Radicis Cortex* Extract on Bone Formation *in Vitro* and *in Vivo*

**DOI:** 10.3390/molecules191219594

**Published:** 2014-11-26

**Authors:** Eunkuk Park, Hyun-Seok Jin, Doo-Yeoun Cho, Jeonghyun Kim, Mun-Chang Kim, Chun Whan Choi, Yilan Jin, Ji-Won Lee, Jin-Hyok Park, Yoon-Sok Chung, Dam Huh, Seon-Yong Jeong

**Affiliations:** 1 Department of Medical Genetics, Ajou University School of Medicine, Suwon 443-721, Korea; E-Mails: jude0815@hotmail.com (E.P.); microchin@hanmail.net (H.-S.J.); danbi37kjh@hanmail.net (J.K.); momotos@hanmail.net (M.-C.K.); jinyilan520@hanmail.net (Y.J.); 2 Department of Biomedical Sciences, Ajou University Graduate School of Medicine, Suwon 443-721, Korea; 3 Department of Family Practice and Community Health, Ajou University School of Medicine, Suwon 443-721, Korea; E-Mail: dooycho@ajou.ac.kr; 4 Clinical Trial Center, Ajou University Medical Center, Suwon 443-721, Korea; 5 Natural Products Research Institute, Gyeonggi Institute of Science & Technology Promotion, Suwon 443-721, Korea; E-Mail: cwchoi78@gmail.com; 6 Korea Food Research Institute, Seongnam 463-746, Korea; E-Mail: dnjs0004@naver.com; 7 Dongwoodang Pharmacy Co., Ltd., Yeongchen 770-864, Korea; E-Mail: navy9376@hanmail.net; 8 Department of Endocrinology and Metabolism, Ajou University School of Medicine, Suwon 443-721, Korea; E-Mail: yschung@ajou.ac.kr

**Keywords:** herbal medicine, osteoporosis, *Lycii Radicis Cortex*, osteoblast, ovariectomized mice, bone mineral density

## Abstract

Osteoporosis is a common skeletal disease caused by decreased bone mass; it enhances the risk of bone fracture. This study aimed to discover novel herbal extract(s) for the treatment of osteoporosis. We screened 64 ethanol extracts of edible plants native to Korea for their ability to increase the cellular proliferation and differentiation of two osteoblastic cell lines: C3H10T1/2 and MC3T3-E1. We selected a* Lycii Radicis Cortex* (LRC), *Lycium Chinese* root bark as the primary candidate. Treatment with LRC extract showed enhanced alkaline phosphatase activity and increased expression of bone metabolic markers *Alpl*,* Runx2*, and* Bglap* genes in both osteoblastic cell lines. There was no effect on the osteoclastic differentiation of primary-cultured monocytes from the mouse bone marrows. Furthermore, the study examined the effect of LRC extract* in vivo* in ovariectomizd (OVX) mice for 8 weeks and 16 weeks, respectively. Bone mineral density (BMD) was significantly higher in LRC extract-administered group than in the non-LRC-administered OVX control group. The results indicated that LRC extract prevented the OVX-induced BMD loss in mice via promoting the differentiation of osteoblast linage cells. These results suggest that LRC extract may be a good natural herbal medicine candidate for the treatment of osteoporosis.

## 1. Introduction

Osteoporosis is a common skeletal disease caused by decreased bone mass. It can lead to increased risk of bone fragility and susceptibility to fracture [1]. The failure of bone homeostasis, due to an increase in osteoclastic bone resorption and a decrease in osteoblastic bone formation, causes the disease [2]. Osteoporosis is a major concern in public health care and the disease has severe consequences if untreated [3,4]. A number of potent pharmacological therapies are available for treatment of osteoporosis. However, reports indicate that some of these drugs have side effects [5]. Recently, osteoporosis management for fracture prevention and risk reduction has moved from monotherapies with antiresorptives or anabolic agents to new combination regimens [6]. In addition, several ingredients, such as calcium, magnesium, vitamin D, vitamin K, phytoestrogen, and prebiotic fiber, are widely used for helping to maintain bone health [7,8,9].

Bone is a complex rigid organ composed of several types of cells undergoing a continuous remodeling process of bone formation and bone resorption followed by the replacement of bone to regulate calcium homeostasis and to repair damaged bones [10]. The two different types of bone cells—osteoblasts that form new bone and osteoclasts that break bone down—both play an important role in the regulation of bone metabolism [10]. A number of factors that either enhance or inhibit bone-remodeling cells control the actions of osteoblasts and osteoclasts, coupled via paracrine signaling. An imbalance in the regulation of bone remodeling between the two processes of bone resorption and bone formation leads to many metabolic bone diseases, including osteoporosis [11].

Herbal treatment for osteoporosis would be advantageous because natural plants have the potential of having fewer side effects, making them more suitable for long‐term use [12]. As potential alternative treatments for osteoporosis, the preventive and therapeutic effects of several natural products derived from plants, including *Carthamus tinctorius*,* Drynaria fortunei*, *Gardenia jasminoides*,* Schizandra chinensis*, and* Ulmus davidiana*, have been reported [13,14,15,16,17]. Reports also indicate that several natural products derived from plants may be beneficial for preventing and treating osteoporosis through the induction of osteoblast differentiation [18].

The aim of this study was to discover herbal extract(s) for effective osteoporosis treatment* in vivo* and* in vitro*. The screening of untested herbal extracts for their effectiveness in increasing osteoblast differentiation and/or proliferation may be an attractive approach to finding new therapeutic osteoporosis drugs that have fewer side effects. This study screened sixty-four ethanol extracts of edible plants native to Korea for their ability to increase the proliferation and differentiation of two osteoblast cell lines. Based on the screening data of both cell lines, *Lycii Radicis Cortex* (LRC) extract emerged as a potential natural-source candidate. We carried out further* in vitro* experiments in cell lines and* in vivo* experiments in the osteoporosis model mice to evaluate the effects of LRC extract on bone formation.

## 2. Results and Discussion

### 2.1. Sixty-Four Plants Native to Korea Were Screened for Cellular Proliferation and Differentiation of Osteoblastic C3H10T1/2 and MC3T3-E1 Cell Lines

Novel therapies under development have mainly focused on anabolic agents to enhance bone strength [19]. The current study aimed to discover the potential natural source(s) having an influence on bone formation. We screened the ethanol extracts of 64 different plants native to Korea for their ability to increase the cellular proliferation and differentiation of two osteoblastic cell lines: C3H10T1/2 and MC3T3-E1. A water-soluble tetrazolium salt (WST) assay provided quantification of cellular proliferation. Alkaline phosphatase (ALP) is a glycoprotein found on the surface of osteoblasts and is a sensitive and reliable indicator of bone metabolism. Hence, the study used an ALP assay as the main method for screening the extracts’ effects on bone formation. We treated three different concentrations (10, 50, and 100 µg/mL) of each plant extract in the osteoblastic cell lines. After three days of incubation with the 64 extracts, they examined cell viability with a WST assay, and determined osteoblastic differentiation levels using the ALP assay (Supplementary Table S1). In each treatment, the experiment was performed independently three times.

Based on the ALP activity and the cell viability of the two cell lines, four herbal extracts that increased ALP activity in both cell lines were selected as primary candidates: *Dipsaci Radix* (50 µg/mL), *Lycii Redicis Cortex* (10 µg/mL), *Radix Achyranthis* (10 µg/mL), and *Puerariae Radix* (10 µg/mL) (Supplementary Table S1). Since reports had already indicated that three plants potentially affected bone metabolism* in vitro* or* in vivo*:* Dipsaci Radix* [20], *Radix Achyranthis* [21], and *Puerariae Radix* [22], the current research focused further study on* Lycii Redicis Cortex* (LRC), a *Lycium Chinense* root bark. LRC is widely used in eastern Asia as a traditional medicine. However, the effect of LRC extract on osteoporosis is unexplored.

### 2.2. LRC Extract Increased Cellular Proliferation and Differentiation of Osteoblast Cell Lines

**Figure 1:**
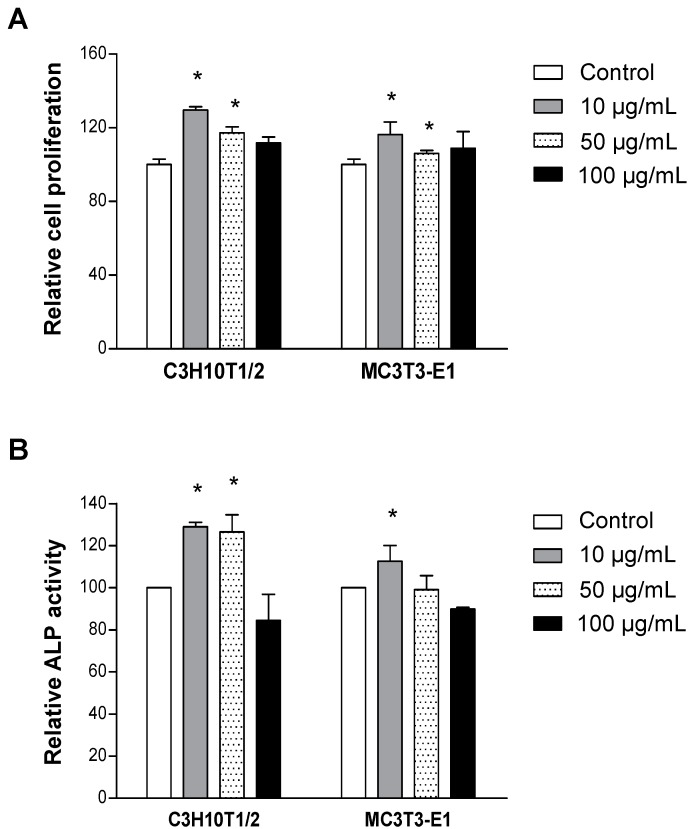
*Lycii Radicis Cortex* (LRC) extract increased cellular proliferation and differentiation in C3H10T1/2 and MC3T3-E1 osteoblastic cell lines. Cells were cultured with three different concentrations of LRC extract (10, 50, and 100 µg/mL) for three days, and cell viability (**A**) and ALP activity (**B**) were analyzed. Control: non-LRC-treated cells. *: *p* < 0.05* vs.* Control.

The study further investigated the anti-osteoporotic effects of LRC extract. First, it examined the effect of LRC extract on osteoblasts. Treatment of 10 µg/mL and 50 µg/mL of LRC extract, respectively, showed significantly increased proliferation in both C3H10T1/2 and MC3T3-E1 cell lines compared to the control group (Figure 1A). The increased ALP activity in both cell lines was observed with 10 µg/mL of LRC extract (Figure 1B). Treatment with a high concentration of LRC extract (100 µg/mL) showed no significant effect on cell proliferation or ALP activity in either cell line. These results indicated that the lowest concentration (10 µg/mL) of LRC extract among those tested was the best condition for osteoblast cell proliferation and differentiation.

**Figure 2:**
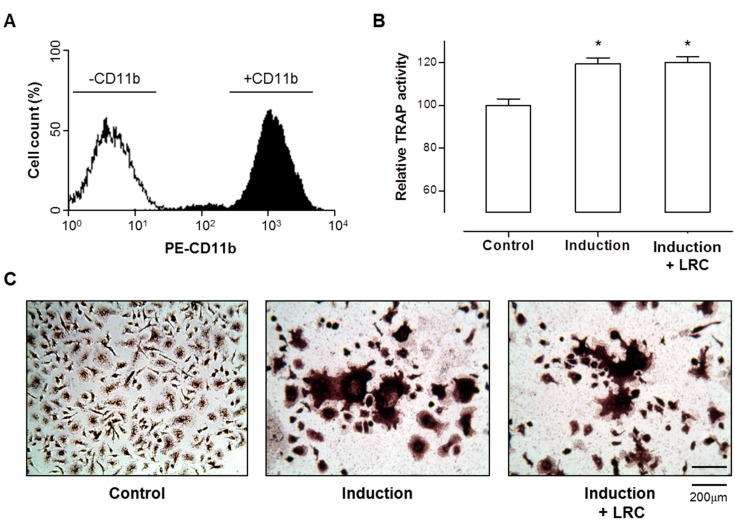
*Lycii Radicis Cortex* (LRC) extract did not affect osteoclastic differentiation. (**A**) Successfully isolated monocytes from mouse bone marrows were identified by immunophenotypic analysis with monocyte-specific surface markers (CD11b antibody) using FACS analysis. (**B**,**C**) Monocyte cells were cultured in the presence of 30 ng/mL of M-CSF and 50 ng/mL of RANKL (Induction), or M-CSF and RANKL with 10 µg/mL of LRC extract (Induction + LRC). Tartrate-resistant acid phosphatase (TRAP) activity assay (B) and TRAP staining (C) measured the differentiated osteoclast cells from mouse bone marrow monocytes. Control: Monocyte cells cultured without M-CSF and RANKL. *: *p* < 0.05* vs.* Control.

Imbalanced bone remodeling is one of the key factors in inducing osteoporosis and many other metabolic bone diseases. Naturally, the daily removal of bone mineral (called resorption) must balance with equal amounts of new mineral deposition, which leads to gradual restructuring of the bone. Therefore, the study examined whether LRC extract had any effect on the differentiation of osteoclast cells, but there was no change in osteoclast differentiation after treatment with LRC extract in the primary-cultured monocytes of mouse bone marrow (Figure 2). These results suggested that LRC extract might promote the proliferation and differentiation of osteoblast cells rather than the inhibition of osteoclastic differentiation.

### 2.3. LRC Extract Increased mRNA Expression of Osteoblastic Markers

**Figure 3:**
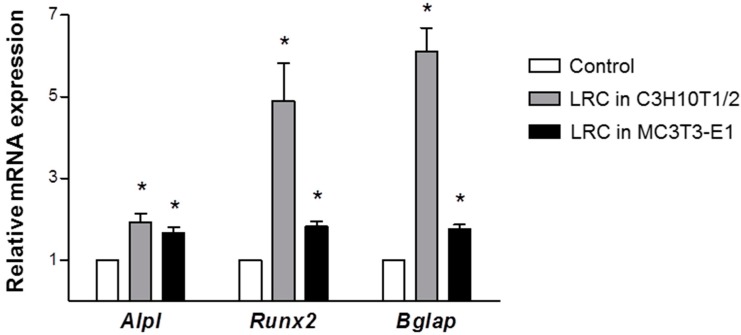
*Lycii Radicis Cortex* (LRC) extract increased the mRNA expression levels of *Alpl*, *Runx2*, and *Bglap* in C3H10T1/2 and MC3T3-E1 osteoblastic cell lines. Cells were treated with 10 µg/mL of LRC extract for three days. The expression level of mRNA was calculated quantitatively by RT-PCR, using targeted gene-specific primers, and then normalized to *Gapdh* mRNA expression. Control: non-LRC-treated C3H10T1/2 or MC3T3-E1 cells. *: *p* < 0.05* vs.* Control.

To confirm further the effect of LRC on the cellular differentiation of osteoblasts, the study investigated changes in the expression of representative osteoblastic marker genes *Alpl* (alkaline phosphatase, ALP), *Runx2* (runt-related transcription factor 2, Runx2), and *Bglap* (bone gamma carboxyglutamate protein, Osteocalcin). Previous studies have suggested the clinical utility of bone remodeling biochemical markers [23]. Either increasing the proliferation of the osteoblastic lineage or inducing osteoblast differentiation can promote bone formation [24]. ALP increases during active bone formation with the induction of osteoblast activity, suggesting that ALP plays a crucial role in the mineralization of newly formed bone [23]. Runx2 is a key transcription factor related to osteoblast differentiation [25]. Osteocalcin, secreted from osteoblasts, is an important factor in the regulation of bone metabolism and in the implication of bone mineralization and calcium ion homeostasis [26]. After treatment with 10 µg/mL of LRC extract for three days in C3H10T1/2 and MC3T3-E1 cell lines, total RNAs were prepared and used as templates for quantitative reverse-transcription PCR (qRT-PCT). There was significantly increased expression of *Alp*, *Runx2*, and *Ocn* in LR-treated cell lines compared to control cell lines (Figure 3). Treatment of LRC extract in MC3T3-E1 cells increased the mRNA expression level of the *Alpl* gene much earlier than it did in those of the *Runx2* and *Bglap* genes (data not shown), as previously reported [27]. This result suggested that LRC extract might stimulate osteoblast differentiation by up-regulating osteoblastic-inducing genes, such as *Alpl*, *Runx2*, and *Bglap*.

### 2.4. LRC Extract Prevented Bone Mineral Density (BMD) Loss in Ovariectomized (OVX) Mice

**Figure 4:**
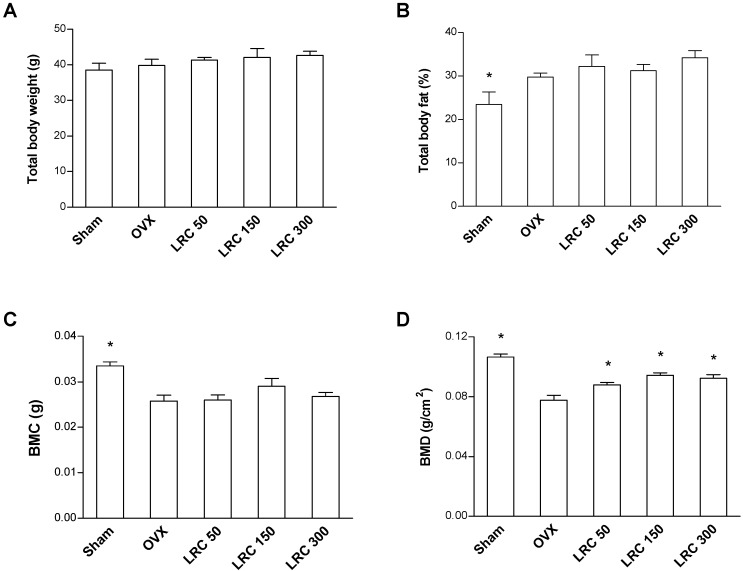
*Lycii Radicis Cortex* (LRC) extract prevented bone mineral density loss in ovariectomized (OVX) mice. The mice received different concentrations of LRC extract (50, 150, 300 mg/kg/day) for 8 weeks. OVX control: non-LRC-administered mice. Their total body weight (**A**), percentage of total body fat (% fat) (**B**), right femur bone mineral content (BMC) (**C**), and right femur bone mineral density (BMD) (**D**) were measured before and after treatment with LRC extract. *: *p* < 0.05* vs.* OVX control.

Based on the study’s* in vitro* results, we further investigated the effect of LRC extract in osteoporosis model animals. OVX mice present with reduced bone mass and quality. In addition, ovariectomies leads to increased body weight in mice with significant decreases in right femur BMD and bone mineral content (BMC) [28]. We first investigated the effects of LRC extract on OVX mice for 8 weeks. Thirty of the 8-week-old female ddY mice underwent either ovariectomy or sham surgery (Sham). The mice were then divided into five groups of six mice each: (1) Sham, (2) OVX control, (3) OVX administrated with 50 mg/kg/day of LRC extract, (4) OVX administrated with 150 mg/kg/day of LRC extract, and (5) OVX administrated with 300 mg/kg/day of LRC extract. After housing the mice for 8 weeks, we compared their total body weight, total body fat percentage, and right femur BMD and BMC. Comparison of the two non-LRC-administered mice groups, the Sham group and the OVX control group, showed increased body fat percentage and decreased BMC and BMD in the OVX group, indicating that the OVX mice were appropriate menopause-induced osteoporosis models (Figure 4). Next, we compared the OVX control and the LRC-administered groups. While body weight, body fat percentage, and BMC were not significantly different between the OVX control and LRC-administered groups, the BMD of the right femur bone was significantly higher in all of the LRC-administered groups (Figure 4). The highest BMD occurred in the 150 mg/kg/day LRC extract-administered group.

**Figure 5:**
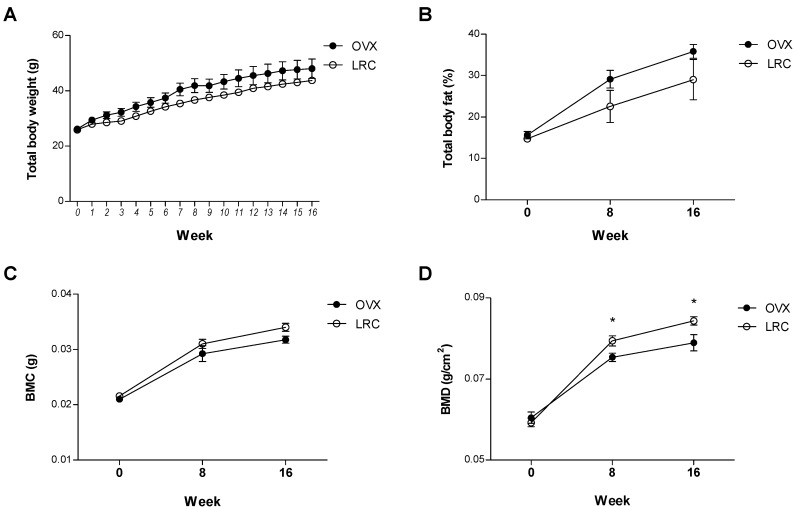
The long-term effects of *Lycii Radicis Cortex* (LRC) extract in ovariectomized (OVX) mice. The mice received LRC extract (150 mg/kg/day) for 16 weeks. OVX control: non-LRC-administered mice. Total body weights (**A**) were measured every week and percentage of total body fat (% fat) (**B**), right femur bone mineral content (BMC) (**C**), and right femur bone mineral density (BMD) (**D**) were measured at 0, 8, and 16 weeks. *: *p* < 0.05* vs.* OVX control.

To investigate the effects of LRC extract* in vivo* for a longer period of time, six mice from the OVX control group and six mice from the LRC extract-administered group (150 mg/kg/day) were housed for 16 weeks. We measured total body weights every week, and body fat percentage, BMC, and BMD of the right femur at zero, 8, and 16 weeks, respectively. Similarly, there were no significant differences between the OVX control and the LRC-administered groups in terms of body weight, body fat percentage, and BMC, although the LRC group showed a slightly lower body weight and fat percentage, and a slightly higher BMC than the OVX control (Figure 5). As expected, BMD was significantly higher in the LRC-administered group than in the OVX control group (Figure 5D). These results indicated that LRC extract prevented BMD loss caused by ovariectomy in mice, and its effect continued for the duration of the test period (16 weeks).

**Figure 6:**
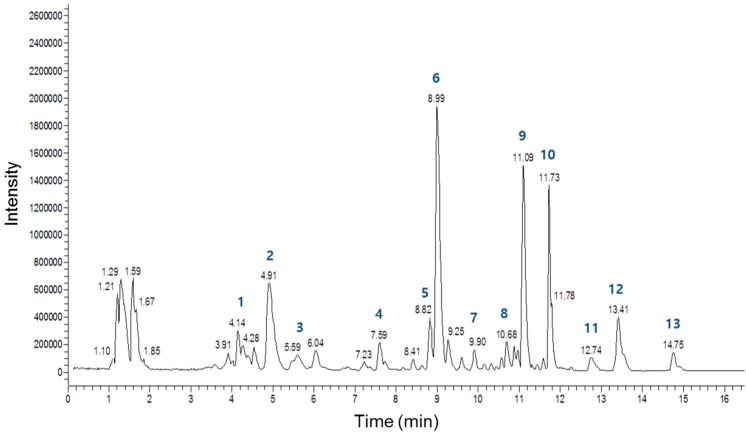
Total ion current chromatogram of the *Lycii Radicis Cortex* (LRC) extract obtained by HPLC-ESI-MS analysis.

**Figure 7:**
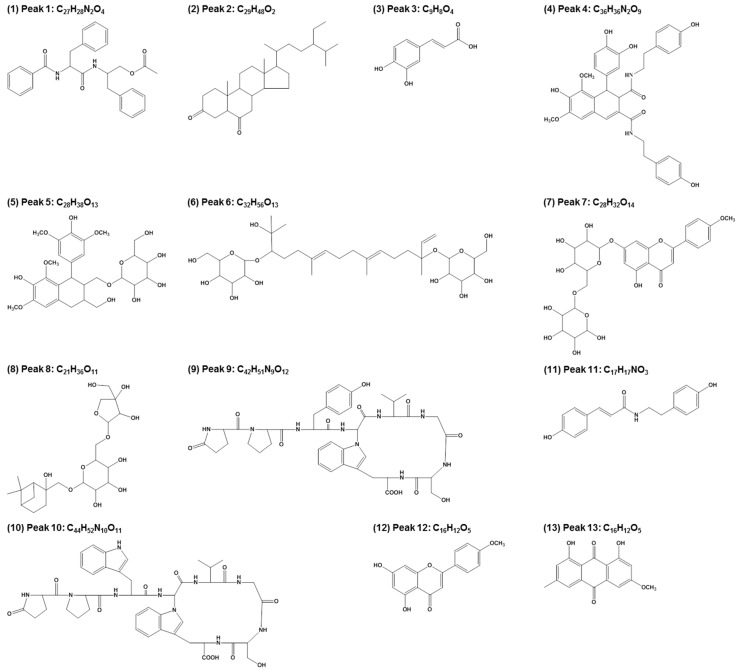
The proposed chemical structures of the 13 main constituents identified in the *Lycii Radicis Cortex* (LRC).

Several studies have demonstrated that LRC extract contains a variety of compounds [29]. To evaluate the main constituents of the LRC extract used in this study qualitatively and quantitatively, we analyzed the extract using a high-performance liquid chromatography-electrospray ionization-tandem mass spectrometry (HPLC-ESI-MS) system. Figure 6 shows a total ion current chromatogram of the LRC extract, and Table 1 summarizes detailed information regarding the main peaks. Overall, 13 major constituents were detected and identified by this analysis, and the most abundant constituents among those analyzed were Lyciumoside III, Lyciumin A, and Lyciumin B. Figure 7 shows the proposed chemical structures of the 13 constituents. Further fractionation and isolation studies should investigate these major constituent(s) and/or other minor constituent(s) responsible for the bone formation effect of LRC extract.

**Table 1:** The main constituents of the *Lycii Radicis Cortex* (LRC) identified by HPLC-ESI-MS analysis.

Peak No.	Compound Name	Molecular Formula	Retention Time	Molecular Weight (*m/z*)
1	Lyciumamide	C_27_H_28_N_2_O_4_	4.14	443.5 [M−H]^−^
2	Stigmastane-3,6-dione	C_29_H_48_O_2_	4.91	472.5 [M+HCOO]^−^
3	Caffeic acid	C_9_H_8_O_4_	5.02	179.1 [M−H]^−^
4	1,2-Dihydro-6,8-dimethoxy-7-hydroxy-1-(3,4-dihydroxyphenyl)-N1,N2-bis[2-(4-hydroxyphenyl) ethyl]-2,3-naphthalene dicarboxamide	C_36_H_36_N_2_O_9_	7.59	685.5 [M+HCOO]^−^
5	Lyoniresinol-3a-*O*-b-d-glucopyranoside	C_28_H_38_O_13_	8.82	627.2 [M+HCOO]^−^
6	Lyciumoside III	C_32_H_56_O_13_	8.99	693.3 [M+HCOO]^−^
7	Linarin	C_28_H_32_O_14_	9.90	637.5 [M+HCOO]^−^
8	(2-Hydroxy-6,6-dimethylbicyclohept-2-yl)methyl 6-*O*-d-apio-β-d-furanosyl-β-d-Glucopyranoside	C_21_H_36_O_11_	10.68	509.3 [M+HCOO]^−^
9	Lyciumin A	C_42_H_51_N_9_O_12_	11.09	872.3 [M+HCOO]^−^
10	Lyciumin B	C_44_H_52_N_10_O_11_	11.73	895.4 [M+HCOO]^−^
11	*trans*-4-Coumaroyltyramine	C_17_H_17_NO_3_	12.74	327.2 [M+HCOO]^−^
12 *	Acacetin	C_16_H_12_O_5_	13.40	329.2 [M+HCOO]^−^
13	Physcion	C_16_H_12_O_5_	14.75	329. 3 [M+HCOO]^−^

* Peak 12 was further confirmed using a purchased Acacetin compound (Sigma-Aldrich).

## 3. Experimental Section

### 3.1. Preparation of Plant Ethanol Extracts

The Korea Promotion Institute for Traditional Medicine Industry (Gyeongsan, Korea) (http://www.kotmin.kr) provided the ethanol extracts of 64 edible plants native to Korea. The plants’ ethanol extracts were prepared based on the previous report [30]. Briefly, after the botanical authentication, a grinding mill turned the dried plant material into powder. Plant powder (100 g) was placed in of 70% aqueous ethanol solution (v/v, 1000 mL) in an occasional shaker at room temperature for 72 h. The extract was then filtered with Whatman No. 2 filter paper to remove debris. The supernatant was collected and concentrated in a rotating evaporator at 40–50 °C and lyophilized under reduced pressure. The dried extract was stored in amber flasks at 4 °C, re-suspended with distilled water, and filtered through a 0.22 µm membrane filter (Sartorius, Gottingen, Germany) before the experiment.

### 3.2. Cell Culture

The murine mesenchymal stem cell line (C3H10T1/2 cells) was purchased from the Korean Cell Line Bank (Seoul, Korea) and grown in a DMEM medium supplemented with 10% fetal bovine serum (FBS), penicillin (100 U/mL), and streptomycin (100 µg/mL). Mouse MC3T3-E1 pre-osteoblast cells were purchased from the RIKEN Cell Bank (Tsukuba, Japan) and grown in a α-MEM medium supplemented with 10% FBS, penicillin (100 U/mL), and streptomycin (100 µg/mL). Cell culture media (DMEM and α-MEM), antibiotics (penicillin and streptomycin), and FBS were purchased from Invitrogen (Carlsbad, CA, USA). Osteoblast differentiation was induced by adding an osteogenic medium containing ascorbic acid (50 µg/mL) and β-glycerophosphate (10 mM) after allowing 24 h for cell adherence (day 0), and the medium was changed every three days. All cultured cells were incubated in a humidified atmosphere at 37 °C and at 5% CO_2_. The study used cells with passages 5–10 (after purchase) for all experiments in both cell lines.

### 3.3. Water-Soluble Tetrazolium Salt (WST) Assay and Alkaline Phosphatase (ALP) Assay

Cells (3 × 10^3^ cells/well) were incubated in a 96-well plate overnight and co-treated with different concentrations of LRC (10, 50, and 100 µg/mL) in the medium for 48 h. An EZ-Cytox Cell Viability Assay Kit (Daeil, Seoul, Korea) tested cell viability. WST (20 µL, 5 mg/mL in PBS) was added to each well, the cells were incubated for another 4 h, and the media were carefully removed. Absorbance was measured at 450 nm and 655 nm using a microplate reader (BioTek; Winooski, VT, USA). ALP activity was measured in total cell lysates after homogenization in a buffer containing 1 mmol/L Tris–HCl (pH 8.8), 0.5% Triton X-100, 10 mmol/L Mg^2+^, and 5 mmol/L p-nitrophenylphosphate as substrates. The absorbance was read at 405 nm (BioTek).

### 3.4. In Vitro Generation of Osteoclasts and Tartrate-Resistant Acid Phosphatase (TRAP) Assay

For primary-cultured monocytes, we flushed bone marrow cells from the femoral bones of 6-week-old mice in the presence of 1 mM ascorbate-2-phosphate (Sigma-Aldrich; St. Louis, MO, USA). Monocyte cells were identified by immunophenotypic analysis with a CD11b antibody (BioLegend; San Diego, CA, USA) using the FACS Aria III cell sorter (BD Biosciences; San Jose, CA, USA) and FACS Diva software (BD Biosciences). Monocyte cells were cultured in the presence of 30 ng/mL M-CSF (PeproTech; Rocky Hill, NJ, USA) and 50 ng/mL RANKL (PeproTech) to induce differentiation to osteoclasts [31]. The differentiated osteoclast cells from monocytes were measured by a TRAP activity assay and staining using the Acid-Phosphatase Kit (Sigma-Aldrich).

### 3.5. Quantitative Reverse-Transcription PCR (qRT-PCR)

Total RNA was extracted from culture cells using a TRIzol reagent (Invitrogen) following the manufacturer’s instructions and quantified by a spectrophotometer (Beckman Coulter; Brea, CA, USA). The extracted RNA were subsequently reverse transcribed using the RevertAid™ H Minus First Strand cDNA Synthesis Kit (Fermentas; Hanover, MD, USA) with the oligo (dT)_15–18_ at a random primer. All real-time RT-PCR measurements were performed using the ABI Prism 7000 Sequence Detection System (Applied Biosystems; Foster City, CA, USA). All PCR amplifications (40 cycles) were performed in a total volume of 25 µL containing 150 ng cDNA using the SYBR Green I qPCR kit (TaKaRa; Shiga, Japan) according to the manufacturer’s recommendations. The specific primers for osteoblast markers were as follows: 5′-TCCCACGTTTTCACATTCGG-3′ and 5′-GGCCATCCTATATGGTAACGGG-3′ for mouse *Alpl*, 5′-TAAAGTGACAGTGGACGGTCCC-3′ and 5′-CCTCAGTGATTTAGGGCGCA-3′ for mouse *Runx2*, and 5′-TAGTGAACAGACTCCGGCG CTA-3′ and 5′-ATGGCTTGAAGACCGCCTACA-3′ for mouse *Bglap.* By normalizing to *Gapdh*, a relative quantification of gene expression was performed using the comparative threshold (Ct) method as described by the manufacturer (Applied Biosystems). The values were expressed as fold change over control. Relative gene expression was displayed as 2^−ΔCt^ (ΔCt = Ct _target gene_−Ct _Gapdh_). Fold change was calculated as 2^−ΔΔCt^ (ΔΔCt = ΔCt _control_−Ct _treatment_).

### 3.6. In Vivo Experiment

The ovariectomized (OVX, n = 30) and sham-operated (Sham, n = 12) 8-week-old female ddY mice were purchased from Shizuoka Laboratory Center Inc. (Hamamatsu, Japan). The mice were acclimated for 10 days prior to experimentation. They were maintained on a diet of Formula-M07 (5.0 g/day) (Feedlab; Hanam, Korea) and tap water (15 mL/day). All mice were housed individually in clear plastic cages under controlled temperature (23 ± 2 °C), humidity (55% ± 5%), and illumination (12 h light/dark cycle). The mice were administered different concentrations of LRC extract: either 50, 150, and 300 mg/kg/day for 8 weeks or 150 mg/kg/day for 16 weeks. Each calculated concentration of LRC extract was added to tap water. The LCR extract-containing water was changed for fresh water every three days, and the volume of LCR extract-containing water was measured every three days for the administered LCR amount. The right femur BMD and BMC and the whole body fat percentage (%) and body weight were measured before and after the administration of LRC extract. The Animal Care and Use Committee of the Ajou University School of Medicine approved the animal research protocol and all experiments were conducted in accordance with the institutional guidelines established by the Committee.

### 3.7. Measurement of Whole Body Fat Percentage and Right Femur BMD and BMC

Whole body fat percentage and right femur BMD and BMC were measured using a PIXImus bone densitometer (GE Lunar; Madison, WI, USA) and calculated by on-board PIXImus software for small animals, adjusted in relation to body weight. After anesthetization using tiletamine/zolazepam (Zoletil) (Virbac Laboratories; Carros, France), the mice were placed on the specimen tray for measurement.

### 3.8. High Performance Liquid Chromatography-Electrospray Ionization-Tandem Mass Spectrometry (HPLC-ESI-MS)

A HPLC analysis was performed using the Thermo Accela UHPLC system (Thermo Fisher Scientific; San Jose, CA, USA), which consists of a quaternary pump, a diode-array detector, an autosampler, and a column compartment. Samples were separated on an ACQUITY BEH C18 column (2.1 mm × 115 mm, 1.7 mm) at room temperature. The mobile phase was composed of acetonitrile (A) and water containing 0.1% formic acid (B). The elution gradient was set as follows: 5% A (0 min), 5% A (3 min), 90% A (15 min) and 100% A (17 min). The mobile phase flow rate was 400 mL/min. An ESI-MS analysis was performed using the Accela liquid chromatographic system (Thermo Scientific) coupled with the LTQ-Orbitrap XL mass spectrometer (Thermo Scientific). The data were collected and analyzed by the Thermo Fisher Xcalibur software package (version 2.2). The mass spectrometer, equipped with an ESI source, was operated in negative ionization mode using the following operating parameters: an electrospray voltage of 4.0 kV, a sheath gas flow rate of 30 arbitrary units, an auxiliary gas flow rate of 8 arbitrary units, a capillary temperature of 275 °C, and a capillary voltage of 30 V. Instrument calibration was performed externally prior to each sequence using a calibration solution. Nitrogen (99.95%) was used as a sheath gas and as an auxiliary gas. The nitrogen served as a collision gas in the HCD cell and as a bath gas in the C-trap.

### 3.9. Statistical Analysis

A statistical software package (SPSS 11.0 for Windows) (SPSS Inc.; Chicago, IL, USA) was used to perform the statistical tests. The statistical significance of differences was assessed by Student’s *t*-test. *p* < 0.05 values were considered significant. Results were expressed as mean ±SEM.

## 4. Conclusions

This is the first report demonstrating the effect of LRC extract on bone formation* in vitro* and* in vivo*. The results of this study demonstrated that LRC extract increased the proliferation and differentiation of osteoblast cells and prevented OVX-induced BMD loss in mice. Notably, in the 16-week* in vivo* experiment, the LRC-administered mice maintained better BMD than the non-LRC-administered OVX control mice for the full test period and showed no deleterious effects on body weight, physical size, morphological characteristics, or diet. This suggests that LRC extract may be a good natural herbal medicine candidate for the treatment of osteoporosis.

## Supplementary Materials

Supplementary materials can be accessed at: http://www.mdpi.com/1420-3049/19/12/19594/s1.
