# Supplementary material for: The Effect of Lycii Radicis Cortex Extract on Bone Formation in Vitro and in Vivo
Source: Molecules. 2014 Nov 26;19(12):19594–609. doi: 10.3390/molecules191219594 (PMC6271141; doi:10.3390/molecules191219594)
Supplement: Supplementary file 1 [file molecules-19-19594-s001.pdf]

Supporting Information

Table S1. Results of water-soluble tetrazolium salt (WST) assay and alkaline phosphatase (ALP) assay of the ethanol extracts of the 64 plants native to Korea in C3H10T1/2 and MC3T3-E1 cells.

No.	Name	Conc. (μg/mL)	C3H10T1/ WST	C3H10T1/ ALP	MC3T3-E1 WST	MC3T3-E1 ALP
1	*Rehmanniae Radix Preparata*	10	.	+	.	.
		50	.	.	.	--
		100	+	.	.	---
2	*Rhizoma Drynariae*	10	.	.	+	-
		50	.	.	.	.
		100	-	--	+	.
3	*Fructus Psoraleae*	10	.	.	.	-
		50	.	-	.	.
		100	--	--	+	-
4	*Acori Graminei Rhizoma*	10	.	+	.	-
		50	.	.	.	-
		100	.	.	.	-
5	*Eucommia Ulmoides*	10	.	.	.	.
		50	.	.	.	-
		100	.	.	+	-
6	*Kalopanacis Cortex*	10	.	.	.	.
		50	+	-	.	-
		100	.	.	.	-
7	*Aralia Continentalis*	10	+	.	.	+
		50	.	.	.	+
		100	.	.	.	-
8	*Carthamus Tinctorius L.*	10	.	-	+	-
		50	--	--	+	--
		100	---	---	.	--
9	*Onion*	10	-	.	+	.
		50	.	.	.	.
		100	.	+	+	.
10	*Rhizoma Cibotii*	10	+	.	.	.
		50	.	-	.	-
		100	-	-	.	--
11	*Codonopsis Pilosula*	10	.	.	.	.
		50	+	.	-	.
		100	.	-	.	-
12	*Cornus Officinalis*	10	.	.	.	.
		50	-	+	.	.
		100	-	-	.	.
13	*Sorbus Commixta*	10	.	-	.	.
		50	--	--	.	-
		100	---	---	+	---
14	*Herba Taraxaci*	10	+	.	.	--
		50	.	.	+	--
		100	-	-	+	--
15	*Cervi Cornu*	10	-	-	-	-
		50	---	---	---	---
		100	---	---	---	---
16	*Saururus Chinensis*	10	--	--	+	--
		50	---	--	+	--
		100	---	--	+	--
17	*Astragali Radix*	10	.	.	+	-
		50	.	.	+	--
		100	-	-	+	--
18	*Chinemys Reevesii (Gray)*	10	.	-	+	-
		50	--	--	++	--
		100	---	---	++	--
19	*Thujae Semen*	10	.	.	+	-
		50	.	-	++	-
		100	-	-	++	--
20	*Radix Achyranthis*	10	+	++	.	++
		50	+	.	+	.
		100	.	.	.	.
21	*Ulmi Cortex*	10	+	+	++	.
		50	+	+	+	-
		100	.	.	.	-
22	*Bombyx Batryticatus*	10	+	+	.	.
		50	.	+	+	-
		100	-	+	+	-
23	*Lithospermi Radix*	10	+	+	.	.
		50	+	+	.	.
		100	+	+	.	-
24	***Lycii Radicis Cortex***	**10**	++	++	+	+
		50	+	++	.	.
		100	+	-	.	-
25	*Nelumbinis Semen*	10	.	+	++	.
		50	.	+	.	.
		100	.	.	.	-
26	*Paeoniae Radix*	10	+	++	.	.
		50	.	+	.	-
		100	.	.	.	-
27	*Morus Alba L.*	10	+	+	+	.
		50	+	.	+	+
		100	++	.	.	.
28	*Rosae Laevigatae Fructus*	10	.	+	.	+
		50	-	.	.	.
		100	--	.	.	.
29	*Herba Cirsii*	10	.	.	+	.
		50	--	--	++	-
		100	---	--	.	--
30	*Ramulus Mori*	10	.	+	+	.
		50	.	.	+	.
		100	-	.	.	--
31	*Mori Folium*	10	.	+	+	.
		50	+	.	.	-
		100	.	.	.	--
32	*Houttuyniae Herba*	10	-	.	.	.
		50	.	.	.	.
		100	.	.	+	.
33	*Radix Sophorae*	10	+	.	.	+
		50	+	.	.	++
		100	+	+	.	.
34	*Rubi Fructus*	10	-	-	.	.
		50	---	---	-	-
		100	---	---	-	--
35	*Herba Artemisiae Annuae*	10	.	+	.	.
		50	.	-	+	.
		100	--	--	+	.
36	*Fagopyrum Esculentum*	10	+	.	+	+
		50	.	.	+	.
		100	--	-	.	-
37	*Semen Torreyae*	10	.	.	+	+
		50	.	-	+	.
		100	--	--	+	--
38	*Puerariae Radix*	10	.	+	.	+
		50	+	.	.	+
		100	+	.	-	-
39	*Artemisiae Argyi Folium*	10	.	.	.	.
		50	.	-	.	.
		100	--	-	.	.
40	*Herba Siegesbeckiae*	10	+	.	+	+
		50	+	-	+	+
		100	.	-	.	-
41	*Albizziae Cortex*	10	--	-	.	.
		50	---	--	-	--
		100	---	---	--	---
42	*Cortex Acanthopanacis*	10	.	.	++	.
		50	.	-	+	.
		100	-	-	.	-
43	*Dipsaci Radix*	10	.	+	.	+
		50	+	++	+	+
		100	-	.	.	.
44	*Salicornia Herbacea*	10	.	.	-	.
		50	+	.	.	.
		100	.	.	.	.
45	*Salicornia Herbacea*	10	+	.	.	.
		50	+	-	.	-
		100	+	-	.	-
46	*Radix Polygoni Multiflori*	10	.	.	.	-
		50	.	.	.	-
		100	-	-	.	-
47	*Scutellaria Baicalensis*	10	-	-	.	.
		50	---	---	.	.
		100	---	---	-	-
48	*Angelicae Gigantis Radix*	10	.	.	.	+
		50	.	.	.	.
		100	+	.	.	-
49	*Curcuma Aromatica Salisb.*	10	.	-	+	.
		50	---	-	++	-
		100	---	--	+	--
50	*Fructus Aurantii Immaturus*	10	+	.	+	-
		50	+	-	+	.
		100	.	-	.	-
51	*Caragana Sinica*	10	.	.	+	-
		50	.	.	+	-
		100	-	.	+	-
52	*Glycine Semen Preparatum*	10	.	.	.	.
		50	.	-	+	.
		100	--	-	+	-
53	*Citrus Unshiu Peel*	10	-	-	.	.
		50	.	.	.	.
		100	.	-	+	-
54	*Aurantii Immatri Pericarpium*	10	.	-	+	.
		50	.	-	+	--
		100	-	-	+	---
55	*Nelumbinis Rhizomatis Nodus*	10	.	.	.	.
		50	-	.	+	-
		100	-	-	+	--
56	*Laminaria Japonica Areschoung*	10	.	.	+	.
		50	.	.	.	-
		100	.	.	.	-
57	*Inonotus Obliquus*	10	-	.	.	-
		50	-	-	.	---
		100	--	-	.	---
58	*Hypsizigus Marmoreus*	10	+	.	.	.
		50	+	.	.	-
		100	.	-	.	-
59	*Aralia Elata*	10	.	.	.	-
		50	.	.	+	-
		100	.	.	.	--
60	*Vitis Vinifera*	10	-	-	++	.
		50	-	.	+	.
		100	.	.	+	.
61	*Prunus Mume*	10	.	.	+	.
		50	.	+	+	.
		100	.	.	++	+
62	*Hovenia Dulcis*	10	.	.	+	.
		50	-	.	+	.
		100	-	.	.	.
63	*Bulbus Allii*	10	.	.	.	+
		50	.	.	.	.
		100	+	+	.	.
64	*Luffa Cylindrica*	10	-	-	+	.
		50	.	.	.	.
		100	.	-	.	-

In each treatment, the experiment was performed independently three times. Symbols: ---, 0~50%; --, 50~70%; -, 70~90%; ., 90~110%; +, 110~130%; ++, 130~150%; +++, 150~ *vs.* non-treated control.
